# Advancing periodontal diagnosis: harnessing advanced artificial intelligence for patterns of periodontal bone loss in cone-beam computed tomography

**DOI:** 10.1093/dmfr/twaf011

**Published:** 2025-02-05

**Authors:** Sevda Kurt-Bayrakdar, İbrahim Şevki Bayrakdar, Alican Kuran, Özer Çelik, Kaan Orhan, Rohan Jagtap

**Affiliations:** Department of Periodontology, Faculty of Dentistry, Eskişehir Osmangazi University, Eskisehir, 26240, Turkey; Division of Oral and Maxillofacial Radiology, Department of Care Planning and Restorative Sciences, University of Mississippi Medical Center School of Dentistry, Jackson, MS, 39216, United States; Division of Oral and Maxillofacial Radiology, Department of Care Planning and Restorative Sciences, University of Mississippi Medical Center School of Dentistry, Jackson, MS, 39216, United States; Department of Oral and Maxillofacial Radiology, Faculty of Dentistry, Eskisehir Osmangazi University, Eskisehir, 26240, Turkey; Department of Oral and Maxillofacial Radiology, Faculty of Dentistry, Kocaeli University, Kocaeli, 41190, Turkey; Department of Mathematics and Computer Science, Faculty of Science, Eskisehir Osmangazi University, Eskisehir, 26240, Turkey; Department of Oral and Maxillofacial Radiology, Faculty of Dentistry, Ankara University, Ankara, 06560, Turkey; Division of Oral and Maxillofacial Radiology, Department of Care Planning and Restorative Sciences, University of Mississippi Medical Center School of Dentistry, Jackson, MS, 39216, United States

**Keywords:** artificial intelligence, periodontal bone defects, tooth segmentation, tooth numbering, CBCT images

## Abstract

**Objectives:**

The current study aimed to automatically detect tooth presence, tooth numbering, and types of periodontal bone defects from cone-beam CT (CBCT) images using a segmentation method with an advanced artificial intelligence (AI) algorithm.

**Methods:**

This study utilized a dataset of CBCT volumes collected from 502 individual subjects. Initially, 250 CBCT volumes were used for automatic tooth segmentation and numbering. Subsequently, CBCT volumes from 251 patients diagnosed with periodontal disease were employed to train an AI system to identify various periodontal bone defects using a segmentation method in web-based labelling software. In the third stage, CBCT images from 251 periodontally healthy subjects were combined with images from 251 periodontally diseased subjects to develop an AI model capable of automatically classifying patients as either periodontally healthy or periodontally diseased. Statistical evaluation included receiver operating characteristic curve analysis and confusion matrix model.

**Results:**

The area under the receiver operating characteristic curve (AUC) values for the models developed to segment teeth, total alveolar bone loss, supra-bony defects, infra-bony defects, perio-endo lesions, buccal defects, and furcation defects were 0.9594, 0.8499, 0.5052, 0.5613 (with cropping, AUC: 0.7488), 0.8893, 0.6780 (with cropping, AUC: 0.7592), and 0.6332 (with cropping, AUC: 0.8087), respectively. Additionally, the classification CNN model achieved an accuracy of 80% for healthy individuals and 76% for unhealthy individuals.

**Conclusions:**

This study employed AI models on CBCT images to automatically detect tooth presence, numbering, and various periodontal bone defects, achieving high accuracy and demonstrating potential for enhancing dental diagnostics and patient care.

## Introduction

The periodontium consists of the periodontal ligament, gingiva, cementum, and alveolar bone that surrounds and supports the teeth. Various conditions such as periodontal diseases and trauma cause damage and loss in these tissues.^1^^,^^2^ The early and accurate detection of periodontal tissue damage with current diagnostic measures and the implementation of a comprehensive follow-up plan in dentistry are of fundamental importance to the development of an effective treatment plan. When deemed appropriate by the periodontist, a range of treatment protocols can be employed to restore periodontal health and preserve tooth function over an extended period of time.^3^

Diagnosis of periodontal diseases is made in line with detailed clinical and radiographic examinations.^4^ There are no radiographic findings in gingivitis cases; however, periodontitis cases progress with irreversible periodontal tissue destruction, attachment loss, and radiographic findings.^5^ Therefore, particularly in diagnosing periodontitis patients, dentists should assess the condition of the alveolar bone through radiographic examination to determine the presence and extent of periodontal bone loss.^6^^,^^7^ Intraoral and extraoral radiographs are used in the determination of periodontal disease. The most preferred ones are periapical, bitewing, and panoramic radiographs that allow us to obtain 2D images.^8^^,^^9^ This may result in the superimposition of anatomical structures, impairing the ability to perform a comprehensive assessment of the 3D osseous structure of the alveolar bone ridge. As the measurement of alveolar bone loss and bone defects is confined to the parasagittal plane, the assessment of conditions that should be evaluated with 3D imaging may be obscured by 2D imaging methods, potentially reducing the sensitivity of the evaluation.^4^^,^^7^^,^^10^^,^^11^ Cone-beam CT (CBCT) is an imaging method used in dentistry for implant planning, orthodontic treatment planning, jaw pathologies, facial trauma, sinuses, and bone examination. Also, it is an inexpensive method and allows 3D imaging.^12^ In addition, with this imaging method, dentists are provided with detailed information and quality images of the jawbones with a limited radiation dose.^13–15^

Factors such as the morphology of the area affected by periodontal disease and the severity of inflammation may cause different types of bone defects. Determinations of these defect types are important for a successful treatment plan.^10^^,^^16^ Numerous studies are showing that periodontal bone defects can be studied and determined successfully using CBCT.^8^^,^^10^^,^^16–22^

In summary, the decision to retain or extract a tooth depends on the type and severity of bone defects.^19^^,^^20^ Treatment protocols such as resective, regenerative, or a combination thereof, including flap surgery, may be chosen based on defect morphology for teeth retained in situ.^23^ Treatment outcomes vary depending on the defect type; for instance, flap surgery and/or resective bone surgery are often preferred for horizontal bone loss, whereas regenerative treatments may be suitable for vertical destructions.^24^ Regenerative treatments typically achieve optimal results in 3-walled vertical defects but exhibit reduced success as the number of intact walls decreases. Combined defects necessitate strategic planning with diverse geometric approaches.^25^

Artificial intelligence (AI) is a computer science that imitates human intelligence.^26^^,^^27^ This branch of science deals with machines capable of analysis, problem-solving, and learning-like human intelligence. The use of AI in the image processing and interpretation phase of diagnosis and treatment planning in the field of health is considered a promising step that will facilitate the work of dentists.^28^^,^^29^ In recent years, both 2D radiographic images in dentistry^30–34^ and 3D radiographic images^27^^,^^31^^,^^35^^,^^36^ have been actively used in the analysis and interpretation by AI. It has been reported to show successful results in many subjects such as caries detection, root fracture detection, root morphology, jaw pathologies, periapical lesion determination, tooth detection, and numbering.^35^^,^^37^^,^^38^ The biggest advantages of these systems are that they facilitate the dentist’s work by acting as a decision-support mechanism in cases such as physician fatigue, intensity, or lack of experience; they also provide practical solutions for reporting.

In the literature, there are a limited number of studies that analyse directly periodontal disease and bone loss types determination using AI systems and convolutional neural network-assisted learning algorithms.^34^^,^^39–43^ To the best of our knowledge, there are no studies in the existing literature that can automatically detect and segment periodontal conditions in CBCT scans. The first aim of this study was to develop an artificial system that can automatically detect teeth and tooth numbers in 3D CBCT scans and to evaluate the success of this system. The second and main goal was to create an advanced deep learning system (DL) that could accurately identify and detect different types of periodontal bone defects.

## Methods

### Study design

**Figure 1.**
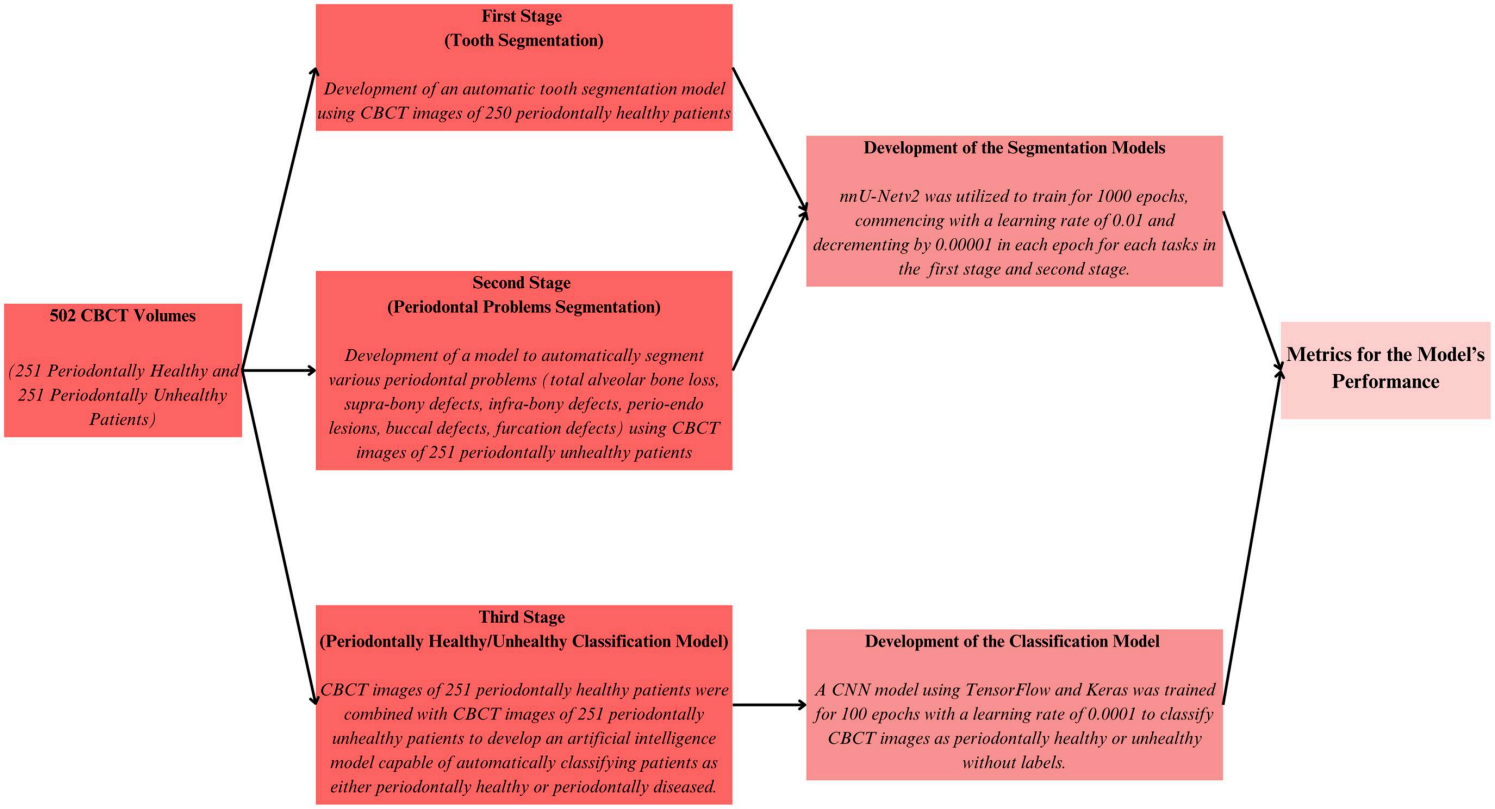
Workflow of the study.

This study was designed as a retrospective study and consisted of 3 stages. A DL system utilizing nnU-Net v2 was created in the first stage to automatically segment and assign numbers to teeth in CBCT volumes. In the second stage, nnU-Net v2-based DL model was constructed to automatically segment different periodontal conditions (total alveolar bone loss, supra-bony defects, infra-bony defects, perio-endo lesions, buccal defects, and furcation defects) in CBCT volumes. After that, TensorFlow and Keras libraries were implemented in the third stage to precisely categorize patients as either periodontally healthy or unhealthy based on CBCT scans without labelling/annotation (Figure 1).

In this study, the Checklist for Artificial Intelligence in Medical Imaging (CLAIM) and Standards for the Reporting of Diagnostic Accuracy Studies (STARD) Checklist were followed for preparing the manuscript. The ethical Committee of Eskişehir Osmangazi University Non-Interventional Clinical Research (Decision date: 20 December 2022, Decision Number: 4, E-25403353-050.99-2300001842) and The University of Mississippi Medical Center (UMMC-IRB-2023-41) approved the study protocol. The study was conducted in accordance with the principles of the Declaration of Helsinki.

### Sample size calculation

The paired 2-sample *t*-test with 251 cases, 95% power, 5% margin of error, and an effect size of dz = 0.20 was determined to have reliable outcomes based on the power analysis performed for estimating the sample size (Figure S1).

### Dataset

This study retrospectively analysed the CBCT archives of Eskisehir Osmangazi University Faculty of Dentistry and the University of Mississippi Medical Centre School of Dentistry to investigate various periodontal issues. The CBCT images stored in the archives were subjected to analysis, and patients presenting with periodontal issues were selected for the development of the DL model. Following that, a group of patients with no periodontal issues in CBCT volumes was selected to construct a cohort of periodontally healthy individuals. All scans selected for the study were from patients over the age of 18 (no ethnic or gender discrimination). Images with dense artefacts, poor-quality images due to patient positioning error or patient movement during acquisition, images that could not be easily selected for the study area, images of patients undergoing orthognathic treatment, and images of patients with bone metabolism disorders showing unusual alveolar bone morphology were excluded.

The complete dataset employed in this study consists of CBCT volumes collected from 502 individual subjects. In the first stage, 250 CBCT volumes were utilized to automatically segment and number teeth. In the second stage, CBCT volumes of 251 patients with periodontal disease were employed to train an AI system to identify different types of periodontal bone defects through segmentation. Lastly, in the third stage, CBCT images of 251 periodontally healthy patients were combined with CBCT images of 251 periodontally unhealthy patients to develop an AI model capable of automatically classifying patients as either periodontally healthy or periodontally diseased.

### Scan acquisition

The CBCT scans used in the study were acquired using Planmeca Promax 3D Mid (OY, Planmeca, Finland) and CS 9600 (Carestream Dental, GA, USA) in various parameters. The patient data are transformed into volumetric data and then exported in the DICOM file format. Therefore, the images can be exhibited in sagittal, coronal, and axial slices. The DICOM files were systematically converted into the Neuroimaging Informatics Technology Initiative (NIfTI) format through a custom-developed script by the CranioCatch Software Team using the Python programming language, with Pydicom, Nibabel, and Opencv libraries.

### Ground truth labelling

In this study, all labelling processes were conducted utilizing the CranioCatch Annotation Tool (CranioCatch, Eskisehir, Turkey). The labelling process began with tooth segmentation and classification according to the FDI system. This process commenced with the segmentation and numbering of the teeth on the axial slices of the CBCT scans. Once the teeth in a CBCT image had been segmented and numbered, the results were reviewed using an interface that allowed for simultaneous viewing of sagittal, axial, and coronal slices. This re-evaluation step helped ensure that no sections were missed and that the numbering was accurate. During this review, some preliminary assessments were performed again to maintain precision.

In the second stage and the third stage, the relevant periodontal problems were evaluated by an expert with over a decade of experience in periodontology (S.K.B.) and experts with over a decade of experience in oral and maxillofacial radiology (I.S.B., R.J.). Specifically, in the second stage of the labelling process, 6 different periodontal conditions were identified and labelled. These were total alveolar bone loss, supra-bony defects, infra-bony defects, perio-endo lesions, buccal defects, and furcation defects. The information contained in these labels is presented in a detailed way in Table S1. The following criteria were taken into account when labelling these parameters.

Total alveolar bone loss is determined in radiographic examinations by establishing a baseline where the healthy periodontium begins 2 mm apical to the cemento-enamel junction. Using this baseline and the crests of the alveolar bone as references, all bone loss around the teeth and surrounding alveolar bone is quantified under the label “total alveolar bone loss.” Defects where the base of the pocket was positioned above the alveolar bone crest, exhibiting horizontal bone loss along the tooth surface, are classified as supra-bony. Conversely, defects where the base of the pocket was within the alveolar bone and demonstrated vertical bone loss are categorized as infra-bony defects. Defects that were found to have direct or indirect communication with the dental pulp are labelled as perio-endo defects. Alveolar bone losses observed on the vestibule or buccal surfaces of the teeth in the form of fenestration or dehiscence are included in the buccal defect category. Bone loss between the roots of multi-rooted teeth is labelled as a furcation defect (Figure S2).

The main investigator demonstrated a high level of consistency in diagnosing and segmenting the parameters over time, as indicated by an Intersection over Union (IoU) >0.90. Additionally, kappa values were calculated to assess the consistency of the observer's decisions regarding supra-bony defects, infra-bony defects, perio-endo lesions, buccal defects, and furcation defects. The kappa values showed excellent agreement when comparing decisions about bone loss characteristics at the 2 different time points for the main investigator, with values ranging from 0.81 to 0.99. After labelling was completed, the dataset was rechecked by 3 experienced oral and maxillofacial radiology investigators (K.O., I.S.B., R.J.). Labels for which no consensus was reached were removed and were not used in algorithm training and outcome measurements.

### Data split

In all models intended for development, 10% of the labelled data was reserved for testing, while 90% was assigned to training. The CBCT volumes used for testing were entirely excluded from the training set, ensuring that the model encountered these images for the first time during the testing phase (Table S1).

For the classification model A total of 502 DICOM CBCT images including 251 periodontal healthy and 251 unhealthy (with periodontal disease) images were converted to NIfTI format. They were divided into 50 tests, 52 validations, and 400 trainings.

### Development of the CNN models

Using Python (v3.6.1; Python Software Foundation, Wilmington, DE, USA), a DL algorithm based on nnU-Net v2 was created for the automatic segmentation of teeth in the first stage and different periodontal situations in the second stage. nnUNetv2 was utilized to train for 1000 epochs, commencing with a learning rate of 0.01 and decrementing by 0.00001 in each epoch. In the third and final stage, a CNN model was developed with the addition of TensorFlow Keras libraries for the purpose of classifying periodontally healthy/unhealthy patients in CBCT images without any labelling. This classification model was trained for 100 epochs with a learning rate of 0.0001 (Appendix S1).

### The nnU-Net v2 workflow

The initial stage of the automatic method configuration of nnU-Net is the fingerprinting of the dataset, which is followed by the execution of heuristic rules. During the development of the model, 3 different U-Net configurations are automatically developed. The first configuration is a 2D U-Net, which performs initial segmentation by matching 2D slices of the images with segmentation masks. This process paves the way for the subsequent use of more complex structures, including 3D U-Net and 3D U-Net cascade, which operate at full resolution. The rapid and efficient pre-processing provided by 2D U-Net enhances the overall performance and adaptability of nnU-Net. The second stage involves a 3D U-Net operating at full image resolution. The final stage involves a cascade of 3D U-Nets, where the first U-Net operates on down-sampled images and the second one is trained to improve the segmentation maps generated by the first one at full resolution. After cross-validation along these lines, the ensemble of different network configurations (including post-processing), determines the best average Dice coefficient (DC) value for the training data. The best configuration will then be used to generate predictions for the test data.

### Metrics for the model’s performance

The confusion matrix method was used as a metric to calculate the success of the model. The true-positive, false-negative, false-positive, and true-negative values were utilized to calculate the metrics of accuracy, precision, sensitivity, and specificity. Also, the statistical evaluation phase of the study was carried out with the receiver operating characteristic (ROC) analysis. The area under the ROC curve (AUC) values are calculated by this analysis. ROC is a probability curve, and the area under it, AUC, represents the degree or measure of separability. As the area under the curve increases, the discrimination performance between classes increases. It is known that as the success of the system increases, the AUC grows and approaches the value of 1. Furthermore, the metrics used to evaluate the performance of the model were DC, 95% Hausdorff Distance (95% HD), and IoU. The model’s performance was deemed successful if the overlap between the predicted and actual values exceeded 50% in every individual image slice.

## Results

### First stage (tooth segmentation)

**Figure 2.**
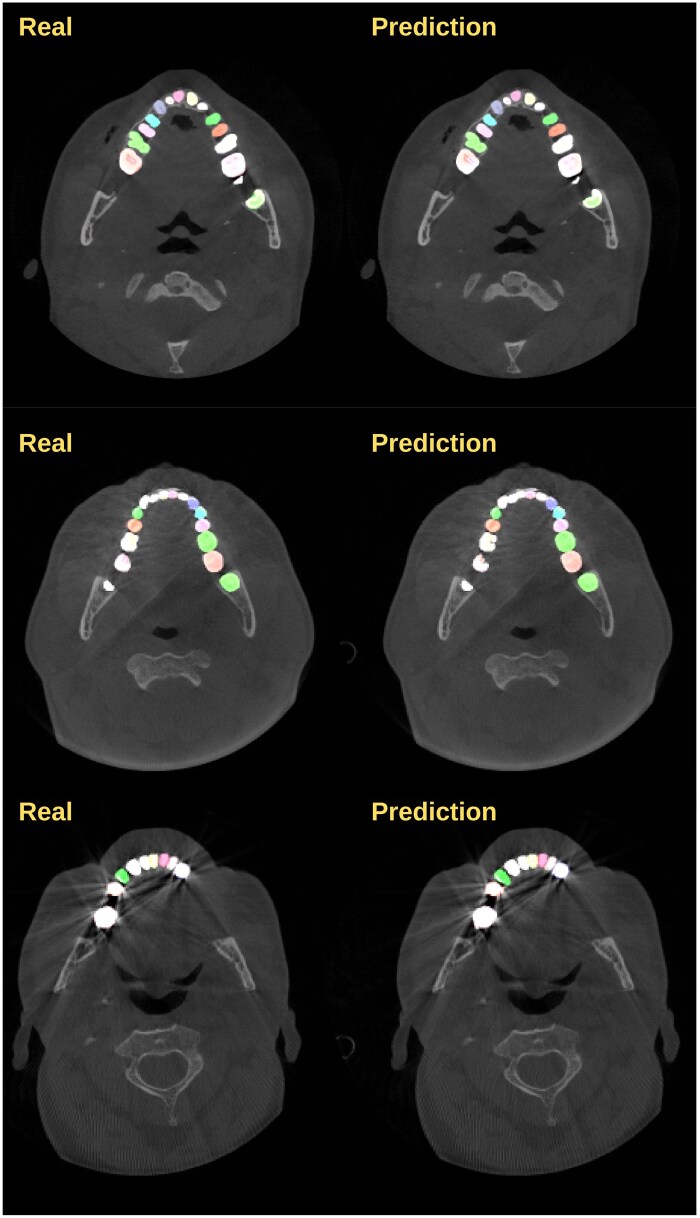
The automatic segmentation of the teeth utilizing the artificial intelligence model in axial CBCT slices.

**Figure 3.**
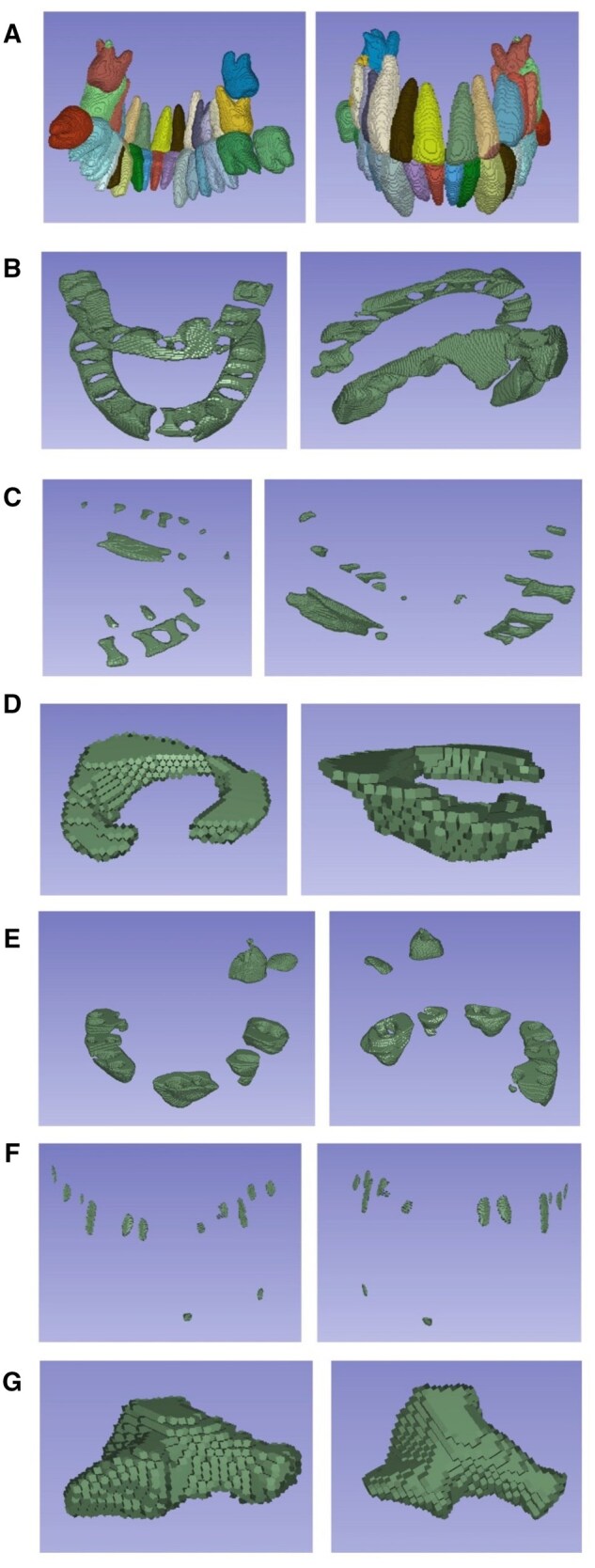
STL images which obtained from the output file given by the artificial intelligence algorithm for the teeth segmenting and periodontal problems segmenting parameter. (A) Teeth segmentation, (B) total alveolar bone loss, (C) supra-bony defects, (D) infra-bony defects, (E) perio-endo lesions, (F) buccal defects, and (G) furcation defects.

**Figure 4.**
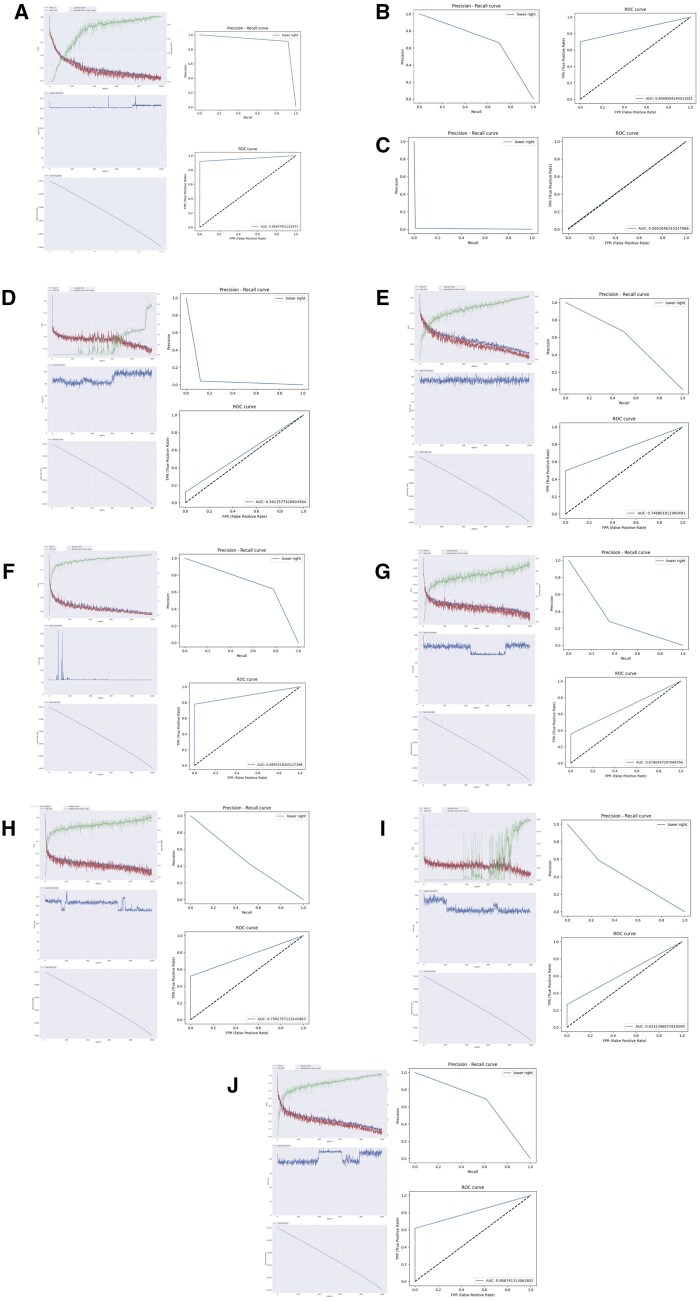
Graphs depicting the performance evaluation results of the artificial intelligence (AI) algorithm developed for detecting various types of periodontal defects. (A) Progress, precision-recall and receiver operating characteristic (ROC) curve graphs for teeth segmentation, (B) Precision-recall and ROC curve graphs for total alveolar bone loss, (C) progress, precision-recall, and ROC curve graphs for supra-bony defects, (D) progress, precision-recall, and ROC curve graphs for infra-bony defects without cropping, (E) progress, precision-recall, and ROC curve graphs for infra-bony defects (training conducted with cropped relevant fields), (F) progress, precision-recall, and ROC curve graphs for perio-endo lesions, (G) progress, precision-recall, and ROC curve graphs for buccal defects without cropping, (H) progress, precision-recall, and ROC curve graphs for buccal defects (training conducted with cropped relevant fields), (I) progress, precision-recall, and ROC curve graphs for furcation defects without cropping, and (J) progress, precision-recall, and ROC curve graphs for furcation defects (training conducted with cropped relevant fields).

The nnU-Net v2-based DL algorithm, designed for automatic tooth segmentation in CBCT volumes, demonstrated an almost perfect accuracy rate of 0.99 in this regard (Figures 2 and 3A). The accuracy, DC, IoU, precision, sensitivity, and specificity values for the teeth in the left upper jaw are 0.99, 0.78, 0.70, 0.77, 0.86, and 0.99, respectively. The values calculated for the teeth in the right upper jaw were 0.99, 0.71, 0.63, 0.78, 0.75, and 0.99, respectively. The measurements for the teeth in the left lower jaw were 0.99, 0.71, 0.63, 0.77, 0.75, and 0.99, respectively. Lastly, the findings for the teeth in the right lower jaw were 0.99, 0.72, 0.64, 0.72, 0.82, and 0.99, respectively (Table 1). The tooth segmentation model's success in segmenting the teeth is detailed for each tooth in Table S2, which includes metrics indicating its performance. Furthermore, AUC value for this model was determined to be 0.95 (Figure 4A).

**Table 1.** The metrics demonstrate the efficacy of the nnU-Net v2 deep learning algorithm in automatically segmenting teeth.

Parameters	Upper left jaw	Upper right jaw	Lower left jaw	Upper right jaw
Accuracy	0.99	0.99	0.99	0.99
Dice score	0.78	0.71	0.71	0.72
Jaccard (IoU)	0.70	0.63	0.63	0.64
Precision	0.77	0.78	0.77	0.72
Sensitivity	0.86	0.75	0.75	0.82
Specificity	0.99	0.99	0.99	0.99

### Second stage (periodontal problems segmentation)

**Figure 5.**
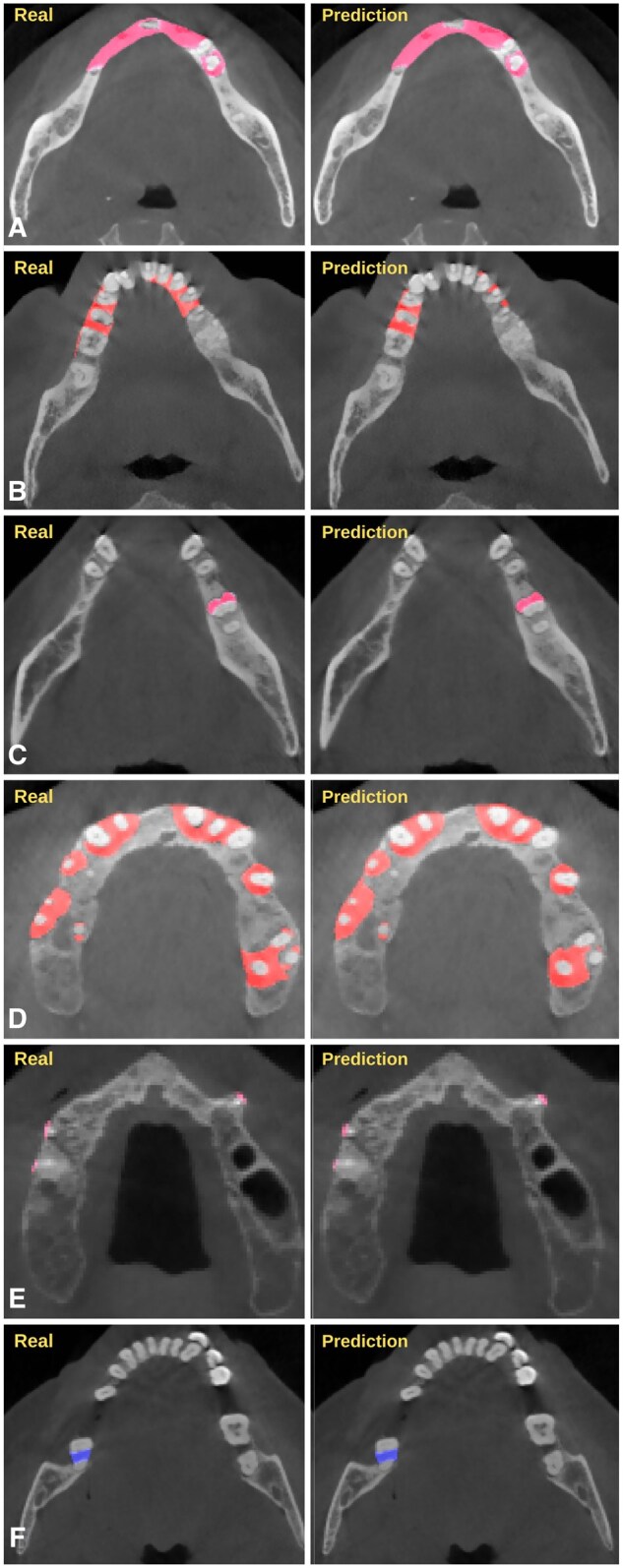
The automatic segmentation of the periodontal problems utilizing the artificial intelligence model in axial cone-beam CT slices. (A) Total alveolar bone loss, (B) supra-bony defects, (C) infra-bony defects, (D) perio-endo lesions, (E) buccal defects, and (F) furcation defects.

The nnU-Net v2-based DL algorithm designed to automatically segment various periodontal disease conditions in CBCT volumes showed an almost perfect accuracy of 0.99 (Figures 3 and 5). In order to evaluate the performance of the AI model developed to automatically segment periodontal problem situations (total alveolar bone loss, supra-bony defects, infra-bony defects, perio-endo lesions, buccal defects, furcation defects), all evaluations were conducted in accordance with the confusion matrix method (Table 2).

**Table 2.** The metrics demonstrate the efficacy of the nnU-Net v2 deep learning algorithm in automatically segmenting various periodontal problems.

Parameters	Total alveolar bone loss	Supra-bony defect	Infra-bony defects	Perio-endo lesions	Buccal defects	Furcation defects
Accuracy	0.99	0.99	0.99	0.99	0.99	0.99
Dice score	0.53	0.36	0.59	0.63	0.45	0.53
95% HD	32.05	63.11	9.29	45.16	32.00	46.50
Jaccard (IoU)	0.38	0.24	0.42	0.47	0.29	0.38
Precision	0.55	0.36	0.81	0.77	0.41	0.62
Sensitivity	0.60	0.43	0.48	0.59	0.51	0.54
Specificity	0.99	0.99	0.99	0.99	0.99	0.99

The AUC values of the models developed for total alveolar bone loss, supra-bony defects, infra-bony defects, perio-endo lesions, buccal defects, and furcation defects were 0.8499, 0.5052, 0.5613, 0.8893, 0.6780, and 0.6780, respectively. No cropping was performed during the development of these models. The models with low AUC values were re-evaluated by cropping. The reason for this was to test whether cropping would increase the success of these models. Accordingly, infra-bony defect, buccal defect, and furcation defect models were retrained with cropping. The AUC values obtained after this process were 0.7488 (without cropping AUC: 0.5613) for infra-bony defect, 0.7592 (without cropping AUC: 0.6780) for buccal defect, and 0.8087 (without cropping AUC: 0.6780) for furcation defect (Figure 4).

### Third stage (periodontally healthy/unhealthy classification model)

The accuracy of the TensorFlow Keras libraries implemented in the CNN model was found to be 80% for healthy individuals and 76% for unhealthy individuals (Figure S3). It was observed that the model was able to identify 20 of 26 healthy patients as healthy and 6 of them as unhealthy. In addition, out of 24 unhealthy patients, 19 of them were found to be unhealthy, and 5 of them were found to be healthy. Furthermore, when the confusion matrix results of the classification training were evaluated, the sensitivity, specificity, precision, accuracy, and F1-score values of the model were 0.76, 0.79, 0.80, 0.78, and 0.78, respectively.

## Discussion

An accurate visualization of the structure of periodontal bone destruction is a critical factor influencing the prognosis of periodontal treatment.^17^ Although 2D radiographs are frequently employed for this imaging purpose, the representation of basic diagnostic details may be compromised due to the attempt to convey the information of the 3D in 2D.^7^ In such instances, CBCT, which provides 3D imaging, is the optimal imaging modality. CBCT imaging has been demonstrated to offer more comprehensive data than periodontal probing and 2D radiography.^11^ In consideration of its numerous advantages, CBCT has gained recognition as a more effective diagnostic tool for use in periodontology.^44^ Nevertheless, due to the higher radiation dose associated with CBCT compared to 2D imaging techniques, it is essential to establish appropriate patient criteria for the utilization of this 3D imaging modality. Bone defects, craters, buccal defects, and furcation involvement are more accurately depicted in CBCT imaging than in 2D radiographs.^45^ However, the interpretation of these tomographic images can be challenging, particularly for those without specialist training. In some cases, important details may be missed due to the high workload and complex workflows of specialized physicians. Computer-aided diagnostic systems can address this challenge by providing support to clinicians, facilitating their work, and enhancing diagnostic capacity.

In the field of dentistry, AI-assisted systems have been frequently employed in the analysis of 2D radiographs. There is a substantial body of research in periodontology that has utilized these systems. In their study, Kim et al^39^ employed the deep convolutional neural networks method on 12 179 panoramic radiographs, developing an AI system to determine periodontal bone resorption. In their study, it was reported that the AI system demonstrated an F1 score of 75%, which was higher than the average success rate of the 5 dental clinicians included in the study (69%). It has been emphasized that the use of AI can lead to more successful results than those achieved by dentists. In a separate study, Krois et al^40^ employed the CNN method to determine periodontal bone resorption in 2001 panoramic radiography. When the results of 6 dentists and AI were compared, it was observed that the accuracy, sensitivity, and specificity were approximately 81%. A study conducted by Kurt-Bayrakdar et al^46^ involved the analysis of 2276 panoramic radiographs, 1137 of which exhibited periodontal bone destruction and 1139 of which demonstrated periodontal health. The researchers achieved a sensitivity of 94%, a specificity of 88%, a precision of 98%, an accuracy of 91%, and an F1 score of 91% in identifying images with periodontal bone destruction. The findings of this study, which employed the Google Net Inception v3 algorithm, demonstrate that AI systems can effectively identify images of bone destruction in 2D panoramic radiography. In their study, Thanathornwong and Suebnukarn^41^ attempted to identify periodontally compromised teeth through the utilization of 100 digital panoramic radiographs and a rapid regional-based convolutional neural network (faster R-CNN) model. The study demonstrated that AI exhibited considerable efficacy, with precision, sensitivity, specificity, and F1 score results reaching 81%, 84%, 88%, and 81%, respectively.

Although a significant number of studies have been conducted using 2D radiographs, the number of studies investigating the diagnosis and classification of CBCT volumes in the field of periodontology remains limited. In their study, Ezhov et al^47^ evaluated the potential of AI to enhance the diagnostic capabilities of dentists. To investigate this, they developed a model that could automatically segment periodontal bone loss and a range of dental conditions in CBCT images. The authors reported that the AI model demonstrated a sensitivity and specificity exceeding 0.90 in the segmentation of periodontal bone loss. Furthermore, the researchers demonstrated that dentists who assess AI-aided CBCT images are more effective in identifying periodontal conditions. However, the authors did not report the DC, 95% HD, and IoU values, which are important parameters for evaluating the success of DL models in segmentation. In addition, the evaluation was limited to bone loss among periodontal conditions, and no AI was developed for automatic segmentation of periodontal bone disorders, such as supra-bony defect, infra-bony defects, perio-endo lesions, buccal defects, and furcation defects. In our study, which comprises 3 stages, the nnU-Net v2-based DL model, developed in the first stage, exhibits 99% accuracy in the segmentation of teeth, with a 73.5% DC overlap with the ground truth. The accurate numbering and segmentation of the teeth in the first stage is of great importance in the interpretation and reporting of periodontal problems in the second stage. Regarding the second stage, in the automatic segmentation of 6 different periodontal conditions, namely total alveolar bone loss, supra-bony defects, infra-bony defects, perio-endo lesions, buccal defects, and furcation defects, the AUC values obtained without any cropping in the images are 0.8499, 0.5052, 0.5613, 0.8893, 0.6780, and 0.6780, respectively. The developed model exhibits a high degree of accuracy in the segmentation of total alveolar bone loss and perio-endo lesions. However, the success of the model was found to be relatively low in other instances of labelling. It is hypothesized that this is because infra-bony defects, buccal defects, and furcation defects are localized and millimetre-scale conditions rather than a generalized condition such as total alveolar bone loss. For this reason, the model was retrained, and its success was re-evaluated by cropping the aforementioned disorders from the labelling. As a result, it was observed that the AUC values increased significantly for the models of infra-bony defect, buccal defect, and furcation defect. In the final stage of the study, a CNN model was developed that was able to distinguish between periodontally healthy and unhealthy individuals without the need for any labelling. The success of this classification model was found to be 80% for healthy individuals and 76% for unhealthy individuals, demonstrating that CNN-based models can effectively distinguish between periodontally healthy and unhealthy patients in CBCT volumes even in the scenario where no labelling is employed.

## Conclusion

The results of our study performed on CBCT images, which provide much more detail and reliability than other imaging systems, show that CNN-based systems can be used in periodontal disease diagnosis and defect typing. It has been found that the AI algorithm developed especially for determining the main defect types is quite successful.

## Supplementary Material

twaf011_Supplementary_Data

## Data Availability

The datasets generated and/or analysed during the current study are not publicly available due to ethical restrictions imposed by the approval conditions from the ethics committee. However, they can be obtained from the corresponding author upon reasonable request.
