# Supplementary material for: Advancing periodontal diagnosis: harnessing advanced artificial intelligence for patterns of periodontal bone loss in cone-beam computed tomography
Source: Dentomaxillofac Radiol. 2025 Feb 5;54(4):268–78. doi: 10.1093/dmfr/twaf011 (PMC12038236; doi:10.1093/dmfr/twaf011)

**SUPPLEMENTARY MATERIAL**

**Advancing Periodontal Diagnosis: Harnessing Advanced Artificial Intelligence for Patterns of Periodontal Bone Loss in CBCT**

**Appendix A**

The XXXXXXXX Faculty of Dentistry’s Dental Artificial Intelligence Laboratory has advanced technology computer equipment including a Dell PowerEdge T640 Calculation Server (Intel Xeon Gold 5218 2.3G, 16C/32T, 10.4GT/s, 22M Cache, Turbo, HT (125W) DDR4-2666, 32GB RDIMM, 3200MT/s, Dual Rank, PERC H330+ RAID Controller, 480GB SSD SATA Read Intensive 6Gbps 512 2.5in Hot-plug AG Drive); PowerEdge T640 GPU Calculation Server (Intel Xeon Gold 5218 2.3G, 16C/32T, 10.4GT/s, 22M Cache, Turbo, HT (125W) DDR4-2666 2, 32GB RDIMM, 3200MT/s, Dual Rank, PERC H330+ RAID Controller, 480GB SSD SATA Read Intensive 6Gbps 512 2.5in Hot-plug AG Drive, NVIDIA Tesla V100 16G Passive GPU); PowerEdge R540 Storage Server (Intel Xeon Silver 4208 2.1G, 8C/16T, 9.6GT/s, 11M Cache, Turbo, HT (85W) DDR4-2400, 16GB RDIMM, 3200MT/s, Dual Rank, PERC H730P+ RAID Controller, 2Gb NV Cache, Adapter, Low Profile, 8TB 7.2K RPM SATA 6Gbps 512e 3.5in Hot-plug Hard Drive, 240GB SSD SATA Mixed Use 6Gbps 512e 2.5in Hot plug, 3.5in HYB CARR S4610 Drive); Precision 3640 Tower CTO BASE Workstation (Intel(R) Xeon(R) W-1250P (6 Core, 12M cache, base 4.1GHz, up to 4.8GHz) DDR4-2666, 64GB DDR4 (4 X16GB) 2666MHz UDIMM ECC Memory, 256GB SSD SATA, Nvidia Quadro P620, 2GB); Dell EMC Network Switch (N1148T-ON, L2, 48 ports RJ45 1GbE, 4 ports SFP+ 10GbE, Stacking).

**Supplementary Material-Table 1.** The data set's contents and the separation

| **Supplementary Material-Table 1. The data set's contents and the separation** | | | |
| --- | --- | --- | --- |
| **Labelling** | **Contained conditions** | **Descriptions** | **Data/Data Split** |
| **Tooth Numbering** |  | The FDI numbering system was used to segment each tooth individually. | Total 250 DICOM files  225 DICOM for training (%90)  25 DICOM for testing (%10) |
| **Total Alveolar Bone Loss** | Horizontal bone loss | It is the form of resorption observed along the length and parallel to the line obtained when the enamel-cement margins of the two adjacent teeth are combined, more apical to the level where the alveolar bone should normally be. | Total 230 DICOM files  207 DICOM for training (%90)  23 DICOM for testing (%10) |
|  | Vertical bone loss | It is the type of angled bone defect that progresses from the enamel cement border to the apical (3 different types according to the number of intact walls).  -1-walled: those with 1 intact wall adjacent to the defect  -2-walled: those with 2 solid walls adjacent to the defect  -3-walled: those with 3 solid walls adjacent to the defect |  |
|  | Combined bone loss | It is a type of bone defect with complex harmony, which contains many defect types together (many defect types such as vertical, 1-walled, 2-walled, and horizontal… are observed together) |  |
|  | Circumferential bone loss | It is the type of bone defect that surrounds the tooth like a funnel or trench. |  |
|  | Crater | It is the type of defect observed if the interdental crest between two teeth is resorbed, but the vertical and lingual wall of the defect continues. |  |
|  | Perio-Endo lesion | If the defect at the root tip is combined with the periodontal defect, that is, it is the name given to the widespread defects extending from the root apical to the enamel-cementum of the tooth. |  |
|  | Furcation defect | They are bone defects extending to the root junctions of multi-rooted teeth;  -Grade 1: Bone losses not exceeding 1/3 of the tooth width  -Grade 2: Bone losses up to 1/3 of the tooth width  -Grade 3: These are furcation defects in which bone loss is observed from one side of the furcation region to the other. |  |
| **Supra-Bony Defect** | - | Only horizontal bone loss defects were included in this group. | Total 138 DICOM files  125 DICOM for training (%90)  23 DICOM for testing (%10) |
| **Infra-Bony Defect** | - | Infra bone defects such as vertical (1-walled, 2-walled, 3-walled) bone loss, combined bone loss, circumferential bone loss, and crater were evaluated under this heading. | ***Without Cropping***  Total 71 DICOM files  64 DICOM for training (%90)  7 DICOM for testing (%10)  ***With Cropping***  Total 63 DICOM files  57 DICOM for training (%90)  6 DICOM for testing (%10) |
| **Perio-Endo Lesions** | - | Perio-Endo lesions are also evaluated separately from total alveolar bone loss group. | Total 39 DICOM files  36 DICOM for training (%90)  3 DICOM for testing (%10) |
| **Buccal Defects** | Fenestration | This is a condition in which the root surface is covered only by the periosteum and gingiva because of the loss of the bone covering on the root surface. | ***Without Cropping***  Total 243 DICOM files  219 DICOM for training (%90)  24 DICOM for testing (%10)  ***With Cropping***  Total 242 DICOM files  218 DICOM for training (%90)  19 DICOM for testing (%10) |
|  | Dehiscence | It is a characteristic oval defect at the cementoenamel junction because of alveolar bone loss, leaving the root exposed. |  |

| **Supplementary Material -Table 2. The metrics demonstrate the efficacy of the nnU-Net v2 deep learning algorithm in automatically segmenting teeth.** | | | | | | | | | |
| --- | --- | --- | --- | --- | --- | --- | --- | --- | --- |
| **Upper Left Jaw** | **Tooth number** | **11** | **12** | **13** | **14** | **15** | **16** | **17** | **18** |
|  | True positive (TP) | 105665 | 92675 | 157192 | 122394 | 135367 | 228799 | 117775 | 45255 |
|  | True negative (TN) | 611333404 | 611374554 | 611278647 | 611381700 | 611375467 | 611264643 | 611358872 | 611487920 |
|  | False positive (FP) | 52339 | 52061 | 84771 | 56809 | 54911 | 66333 | 51935 | 42631 |
|  | False negative (FN) | 86263 | 58381 | 57061 | 16768 | 11926 | 17896 | 49089 | 1865 |
|  | Accuracy | 0.9992 | 0.9993 | 0.9992 | 0.9997 | 0.9997 | 0.9998 | 0.9999 | 0.9999 |
|  | Dice score | 0.7866 | 0.8045 | 0.8473 | 0.7699 | 0.7762 | 0.8792 | 0.8055 | 0.5916 |
|  | 95% HD | 6.8360 | 12.0527 | 17.57893 | 18.6361 | 21.0160 | 41.9594 | 49.8785 | 52.0869 |
|  | Jaccard (IoU) | 0.7060 | 0.7200 | 0.7651 | 0.6998 | 0.7113 | 0.7992 | 0.7019 | 0.5007 |
|  | Precision | 0.8193 | 0.8320 | 0.8413 | 0.7606 | 0.7598 | 0.8487 | 0.7990 | 0.5397 |
|  | Sensitivity | 0.7683 | 0.7862 | 0.8563 | 0.8933 | 0.9125 | 0.9363 | 0.8452 | 0.9260 |
|  | Specificity | 0.9997 | 0.9996 | 0.9995 | 0.9997 | 0.9997 | 0.9999 | 0.9999 | 0.9999 |
| **Upper Right Jaw** | **Tooth number** | **21** | **22** | **23** | **24** | **25** | **26** | **27** | **28** |
|  | True positive (TP) | 88388 | 55509 | 112899 | 106536 | 103180 | 196772 | 177036 | 96744 |
|  | True negative (TN) | 611353656 | 611414754 | 611338849 | 611378793 | 611425794 | 611302595 | 611294424 | 611428268 |
|  | False positive (FP) | 44606 | 31418 | 25139 | 29420 | 10324 | 18388 | 49438 | 11189 |
|  | False negative (FN) | 91021 | 75990 | 100784 | 62922 | 38373 | 59916 | 56773 | 41470 |
|  | Accuracy | 0.9992 | 0.9994 | 0.9993 | 0.9996 | 0.9999 | 0.9998 | 0.9998 | 0.9999 |
|  | Dice score | 0.7939 | 0.7386 | 0.6869 | 0.6132 | 0.7973 | 0.6850 | 0.7779 | 0.6617 |
|  | 95% HD | 6.8242 | 15.4790 | 37.1970 | 31.1562 | 17.7209 | 23.1134 | 26.1951 | 13.9851 |
|  | Jaccard (IoU) | 0.7131 | 0.6444 | 0.6141 | 0.5336 | 0.6995 | 0.6313 | 0.6703 | 0.5934 |
|  | Precision | 0.8181 | 0.7830 | 0.6945 | 0.6147 | 0.8835 | 0.7794 | 0.8338 | 0.8589 |
|  | Sensitivity | 0.7819 | 0.7265 | 0.7818 | 0.7028 | 0.7531 | 0.7873 | 0.7844 | 0.6932 |
|  | Specificity | 0.9997 | 0.9998 | 0.9999 | 0.9999 | 0.9999 | 0.9999 | 0.9999 | 0.9999 |
| **Lower Right Jaw** | **Tooth number** | **31** | **32** | **33** | **34** | **35** | **36** | **37** | **38** |
|  | True positive (TP) | 72426 | 83185 | 128052 | 79784 | 56935 | 113281 | 134147 | 105043 |
|  | True negative (TN) | 611423034 | 611429260 | 611355303 | 611382221 | 611380245 | 611171355 | 611358278 | 611390977 |
|  | False positive (FP) | 34241 | 12890 | 18775 | 35735 | 37905 | 71045 | 15036 | 39168 |
|  | False negative (FN) | 47970 | 52336 | 75541 | 79931 | 102586 | 221990 | 70210 | 42483 |
|  | Accuracy | 0.9995 | 0.9996 | 0.9995 | 0.9995 | 0.9994 | 0.9990 | 0.9999 | 0.9998 |
|  | Dice score | 0.7931 | 0.7858 | 0.8090 | 0.7064 | 0.6250 | 0.5946 | 0.7895 | 0.5925 |
|  | 95% HD | 5.6194 | 6.6688 | 10.4124 | 32.6132 | 45.1385 | 44.0693 | 7.7470 | 40.2432 |
|  | Jaccard (IoU) | 0.7166 | 0.7080 | 0.7458 | 0.6283 | 0.5566 | 0.5251 | 0.7159 | 0.5171 |
|  | Precision | 0.7849 | 0.8711 | 0.8850 | 0.7962 | 0.7072 | 0.66436 | 0.8965 | 0.6023 |
|  | Sensitivity | 0.8060 | 0.8070 | 0.8378 | 0.7259 | 0.6535 | 0.6122 | 0.7834 | 0.8366 |
|  | Specificity | 0.9998 | 0.9999 | 0.9999 | 0.9999 | 0.9999 | 0.9999 | 0.9999 | 0.9998 |
| **Lower Left Jaw** | **Tooth number** | **41** | **42** | **43** | **44** | **45** | **46** | **47** | **48** |
|  | True positive (TP) | 71992 | 79855 | 123008 | 82136 | 47183 | 99483 | 121306 | 131439 |
|  | True negative (TN) | 611429547 | 611433890 | 611363253 | 611372898 | 611388398 | 611195741 | 611376175 | 611389303 |
|  | False positive (FP) | 47007 | 52379 | 75077 | 86835 | 103332 | 213795 | 69489 | 38290 |
|  | False negative (FN) | 29125 | 11547 | 16333 | 35802 | 38758 | 68652 | 10701 | 18639 |
|  | Accuracy | 0.9995 | 0.9996 | 0.9995 | 0.9995 | 0.9994 | 0.9991 | 0.9999 | 0.9999 |
|  | Dice score | 0.7905 | 0.7710 | 0.8028 | 0.6873 | 0.6250 | 0.5798 | 0.7817 | 0.7278 |
|  | 95% HD | 5.9726 | 10.1662 | 8.5584 | 15.3388 | 28.2463 | 63.3374 | 28.0067 | 50.4796 |
|  | Jaccard (IoU) | 0.7128 | 0.6890 | 0.7374 | 0.6178 | 0.5482 | 0.5057 | 0.7191 | 0.6512 |
|  | Precision | 0.7954 | 0.7961 | 0.8223 | 0.7007 | 0.6721 | 0.5599 | 0.7759 | 0.7126 |
|  | Sensitivity | 0.7878 | 0.8586 | 0.8893 | 0.7788 | 0.7148 | 0.7435 | 0.9197 | 0.8962 |
|  | Specificity | 0.9997 | 0.9996 | 0.9995 | 0.9995 | 0.9994 | 0.9991 | 0.9999 | 0.9999 |

**Supplementary Material-Figure 1.** Power analysis graph illustrating the calculation of sample size.


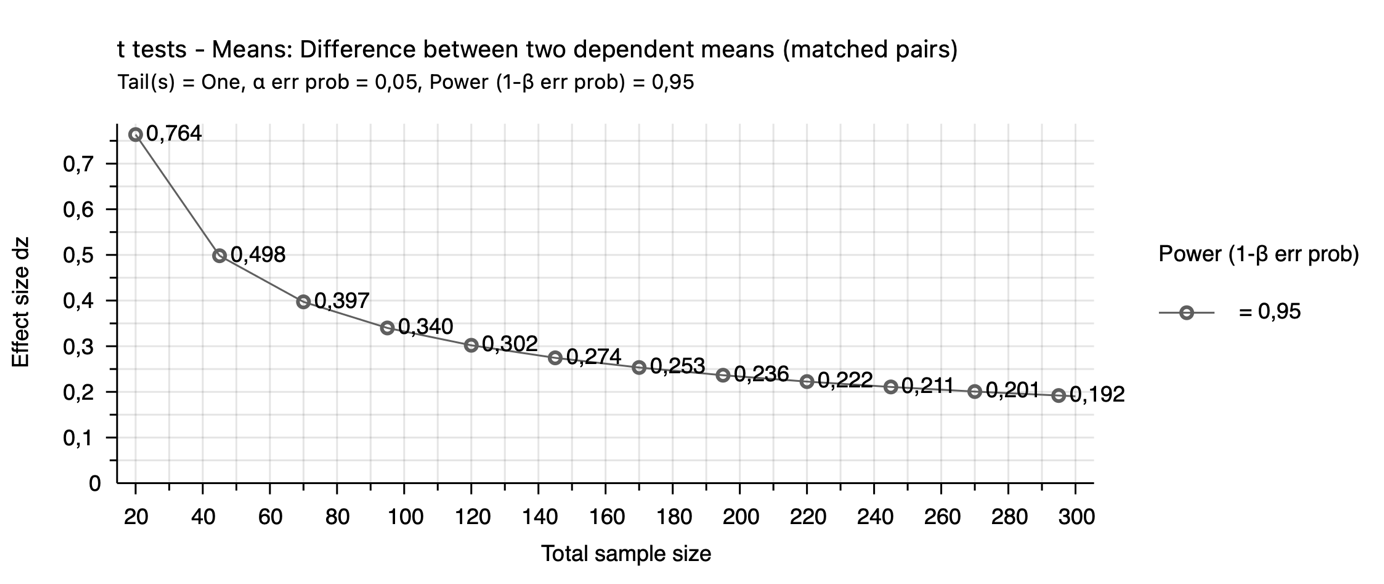


**Supplementary Material-Figure 2**. A) Sagittal, coronal, and axial section views of a patient with completed tooth numbering labeling. B) Sagittal, coronal, and axial section views of a patient with completed periodontal defect labeling.


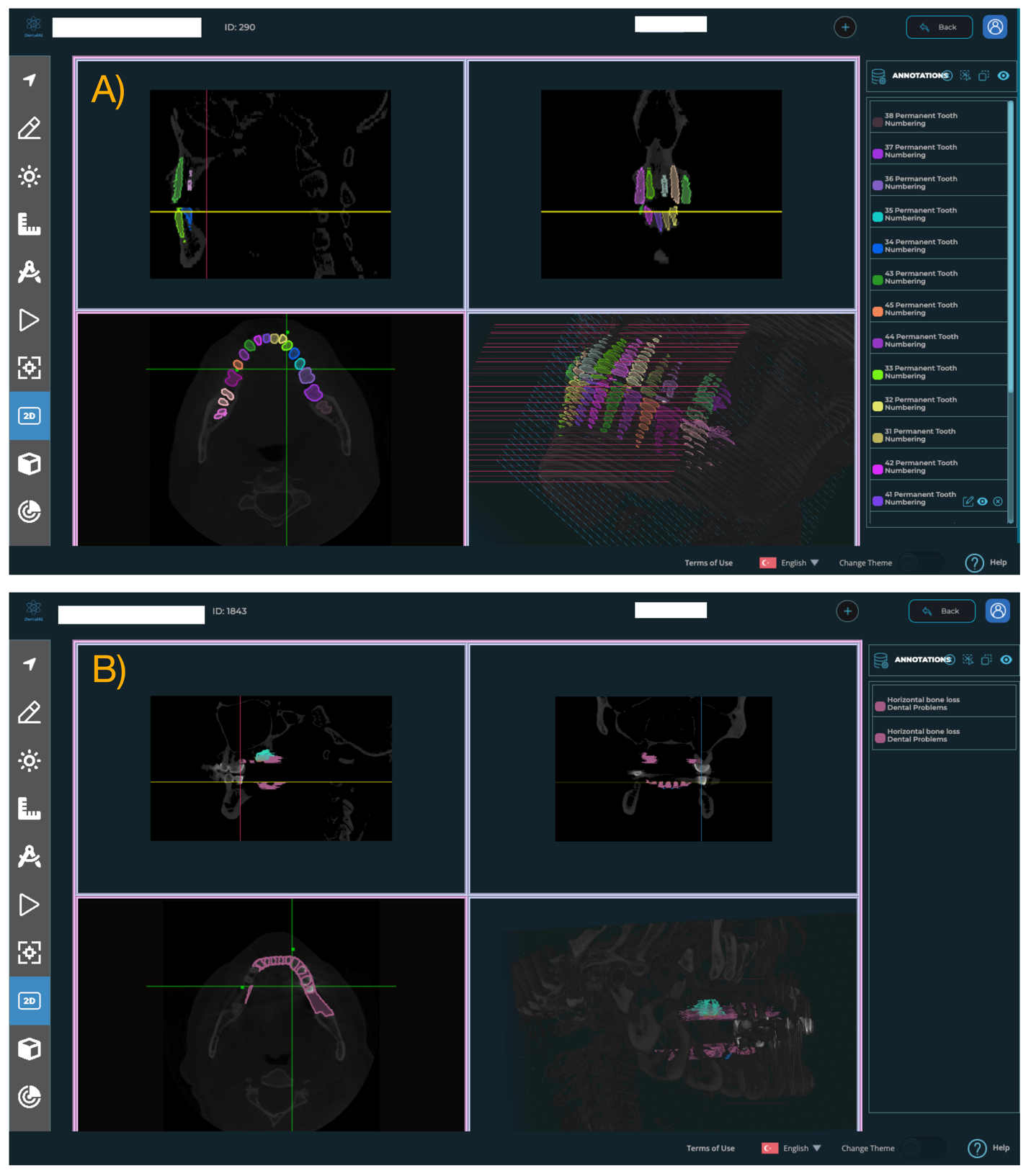


**Supplementary Material-Figure 3**. Model accuracy was found 80% for healthy and 76% for unhealthy.


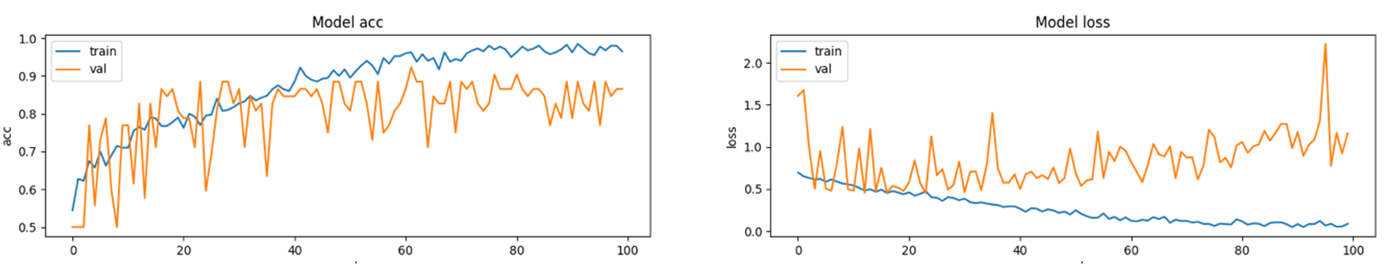

Supplement: twaf011_Supplementary_Data [file twaf011_supplementary_data.docx]
